# BioSig3D: High Content Screening of Three-Dimensional Cell Culture Models

**DOI:** 10.1371/journal.pone.0148379

**Published:** 2016-03-15

**Authors:** Cemal Cagatay Bilgin, Gerald Fontenay, Qingsu Cheng, Hang Chang, Ju Han, Bahram Parvin

**Affiliations:** 1 University of Nevada, Reno, Department of Electrical and Biomedical Engineering, Reno, NV, United States of America; 2 Lawrence Berkeley National Laboratory, Life Sciences Division, Berkeley, CA, United States of America; Pennsylvania State Hershey College of Medicine, UNITED STATES

## Abstract

BioSig3D is a computational platform for high-content screening of three-dimensional (3D) cell culture models that are imaged in full 3D volume. It provides an end-to-end solution for designing high content screening assays, based on colony organization that is derived from segmentation of nuclei in each colony. BioSig3D also enables visualization of raw and processed 3D volumetric data for quality control, and integrates advanced bioinformatics analysis. The system consists of multiple computational and annotation modules that are coupled together with a strong use of controlled vocabularies to reduce ambiguities between different users. It is a web-based system that allows users to: design an experiment by defining experimental variables, upload a large set of volumetric images into the system, analyze and visualize the dataset, and either display computed indices as a heatmap, or phenotypic subtypes for heterogeneity analysis, or download computed indices for statistical analysis or integrative biology. BioSig3D has been used to profile baseline colony formations with two experiments: (i) morphogenesis of a panel of human mammary epithelial cell lines (HMEC), and (ii) heterogeneity in colony formation using an immortalized non-transformed cell line. These experiments reveal intrinsic growth properties of well-characterized cell lines that are routinely used for biological studies. BioSig3D is being released with seed datasets and video-based documentation.

## Introduction

The current trend in high content screening (HCS) has been the utilization of more complex model systems that mimic both structural and functional properties of cells *in vivo* [1]. C. elegan and zebra fish are examples of complex model systems currently being used to study biological processes and development [2–4]. Complex model systems have been the main motivation behind the development of imaging bioinformatics systems for phenotypic screening [5], with the main drivers for the utility of these biological systems being high fecundity and a short life span. Similarly, 3D cell culture models have emerged as effective systems to study tumor initiation, biological processes, and reversion properties with respect to therapeutic targets. In contrast to 2D cell culture models, 3D cell culture models (i) have different patterns of development and can be functional, (ii) respond differently to therapeutic targets [6], and (iii) have different patterns of gene expression [7]. Hence, the 3D cell culture model is a potential model system to bridge between the 2D model systems and the animal studies. In some systems, 3D colonies are imaged using elegant specialized microscopy and profiled using 2D image analysis and global intensity measurements [6, 8, 9]. In this manuscript, 3D cell culture systems are imaged in 3D, using confocal microscopy, and processed in 3D volume to profile every nucleus in the colony and colony organization. Colony organization is an important index for classification of normal and aberrant cell lines [10–13], and, in many cases removes the need for a more expensive assay optimization and labeling for specific molecular endpoints. Although screening, based on phase contrast imaging can be informative in some cases, however, it is not sufficient. For example, the non-transformed human mammary cell line of MCF10A has a similar signature to cell line model of the ductal in situ carcinoma (DCIS), MCF7 cell line, [12] when imaged in 2D, using phase contrast microscopy. Both MCF10A and MCF7 organize themselves as a sphere in 3D; however, MCF10A is organized as a hollow sphere (usually referred to as lumen forming), while MCF7 is organized as a solid sphere. Therefore, imaging, visualization, and analysis of the 3D colonies are required and need to be performed in full 3D volume. Furthermore, screening based on colony formation is low cost and can be part of a primary screen prior to any additional subcellular or molecular endpoints. This is one of the gaps that BioSig3D is designed to overcome. However, high content screening of the cell culture models also has a large number of experimental variables, which need to be associated with computed quantitative information. These variables include cell lines, therapeutic targets, concentration of therapeutic targets, and harvest time. As a result, an integrated system has to link factors associated with the perturbation of the microenvironment, with the 3D colony organization and relevant molecular end points. In other words, a simple analytical capability is insufficient, and an end-to-end solution is needed to address the complexities of a high content screening assay. BioSig3D is the first web-based system to address relevant issues associated with high content screening of 3D cell culture models, which have been imaged in 3D using either confocal or deconvolution microscopy. Table 1 compares BioSig3D with several research platforms, such as Open Microscopy Environment (also known as OMERO) [14], the cell profiler [15], Vaa3D [16], and ACME [17]. BioSig3D shares some of the goals of the OMERO, but the focus is on 3D cell culture models that have been imaged in full 3D volume. As a result, colonies need to be processed in 3D, rendered in 3D for quality control, and distinct features of colony organization need to be captured for bioinformatics analysis. BioSig3D builds on our earlier systems [18] and lessons learned from similar efforts for building imaging bioinformatics systems.

**Table 1:** Comparison of BioSig3D with other systems.

**Comparison of the System Architecture from a User’s Perspective**								
**Systems**		**Web-based**	**control vocabulary support, database integration**		**Integrated experimental and resource management**	**Remote collaboration and access control**		**High-content screening in 3D**
**BioSig3D**		Yes	Yes		Yes	Yes		Yes
**Cell profiler**		No	No		No	No		No
**Open Microscopy Environment (OMERO)**		Yes	Partially		Partially	Yes		No
**Vaa3D**		No	No		No	No		No
**ACME**		No	No		No	No		Yes
**Comparison of the Analytical and Bioinformatics Modules**								
**Systems**		**Full 3D volumetric analysis**	**Advanced image analysis algorithms (Tables 2 and 3)**		**Multiparametric profiling of colony organization**	**Correlative analysis between computed nuclear and colony organization indices**		**Mean (heatmap) and heterogeneity (subtyping)**
**BioSig3D**		Yes	High		Yes	Yes		Yes
**Cell profiler**		No	Medium (only 2D analysis)		No	No		Partially
**OMERO**		No	Low (only 2D analysis)		No	No		No
**Vaa3D**		Yes	Low		No	No		No
**ACME**		Yes	Medium (extensive integration of existing methods)		No	No		No
**Comparison of Visualization Modules**								
**Systems**	**Web-based 3D volumetric visualization**		**Web-based 3D surface rendering based**	**Thumbnails of a single slice and/or serial sections**				
**BioSig3D**	Yes		Yes	Yes				
**Cell profiler**	No		No	Yes				
**OMERO**	No		No	Yes				
**Vaa3D**	No		No	No				
**ACME**	No		No	No				

The ultimate goal of BioSig3D is not just segmentation, visualization 3D spatial image-based data, and quantitative analysis of cellular features, which has been the focus of a number of systems [16, 17]. Here, the goal is to design and execute high content screening experiments with 3D cell culture models, where *3D organization*, derived from nuclear segmentation, is an important endpoint for quantifying aberrant phenotypes. Another important facet for profiling 3D colonies is the presence of heterogeneous colony organization, and, in sensitive assays, colony heterogeneity may be an important endpoint. BioSig3D also enables heterogeneity analysis for each computed index or collection of indices. Two experiments have been designed to study colony formation using either on top or embedded 3D cell culture models [19]. The first examines colony morphogenesis for a panel of human mammary epithelial cell (HMEC) lines of non-transformed and malignant origins using the on top culture method, and the second experiment utilizes embedded 3D cell culture model to profile heterogeneity in colony formation, where heterogeneity is expressed as a function of the frequencies of subtypes (e.g., clusters in a dataset). Subsequently, populations associated with each subtype can also be rendered in 3D. The concept of colony heterogeneity, in 3D cell culture model, has rarely been addressed in the past, but it is an important observation since an index to heterogeneity can improve readouts for sensitive assays. Implementation of heterogeneity analysis, in BioSig3D, is a form of content-based retrieval [20, 21], where retrieval is based on clusters of colonies that represent a unique phenotype. These baseline experiments will be complementary to the design and verification of BioSig3D, and enables researchers to design new experiments. BioSig3D is made publicly available, at biosig.lbl.gov/BioSig3D, as a VMWare which will require no complex installation of various third party packages. The manuscript includes three components of (i) materials and methods, (ii) components of BioSig3D that presents the systems software architecture, and (iii) results and discussion, which provides case studies on the utility of the system.

## Materials and Methods

### Sample preparation and microscopy

#### Embedded 3D culture

Immortalized human mammary primary cells (HMEC) line of MCF10A were obtained from ATCC, and cultured in 1.0% agarose (Sigma) gels supplemented with Matrigel matrices (BD). The embedded cell culture protocol consists of: (i) 8,000 cells carefully suspended in 40 μL Matrigel and stored on ice; (ii) 40 μL of 2.0% agarose solution is added to the cell suspension and mixed well; (iii) the mixture is placed in an 8-well chambered coverglass (Nunc Lab Tek II) and incubated for 20 minutes to form a homogenized gel in the incubator, before adding 500μL growth medium per well (DMEM/F12 supplemented with 5% horse serum, 20 ng/mL EGF, 0.5 μg/mL hydrocortisone, 100 ng/mL cholera toxin, 10μg/mL insulin and 1% penicillin streptomycin); (iv) the temperature is maintained at 37°C and is supplemented with 5% CO_2_; and (v) the medium is changed every 3 days. For “on top” cell culture protocol, see [11, 22]. The growth medium recipe was DMEM/F12, supplemented with 5% horse serum, 20 ng/mL EGF, 0.5 μg/mL hydrocortisone, 100 ng/mL cholera toxin, 10μg/mL insulin and 1% penicillin streptomycin.

#### Immunofluorescent staining

Cells were fixed at room temperature in 10% formalin for 30 minutes, within the hydrogel. After 3 cycles of PBS washes, cells were permeabilized using a 0.1% Triton X-100 solution for 5 minutes. After another 3 cycles of PBS wash, the nuclei were stained with 4’-6-diamidino-2-phenylindole (50 ng/mL in PBS) (DAPI, Life Science Technology).

#### Microscopy

Stained samples were visualized using a Zeiss LSM 710 system equipped with a Zeiss Apochromat 40X/1.1 (0.8 mm WD) objective lens. The 405 nm mercury laser, with an intensity of 1%, was used to excite DAPI. The digital gain was adjusted to approximately ¾ of the maximum gain, which kept the dynamic range of the pixel between 500–2000 (12 bits). The 3D stack function of the *Zen software* was used to collect raw 3D information for each colony, and was concurrent with “Z correction” of the pixel intensity of thick samples. The voxel size was set to 0.25μm×0.25μm×1μm. The collected data were saved as lsm files and uploaded to BioSig3D.

### Components of BioSig3D

#### BioSig3D services

**Fig 1:**
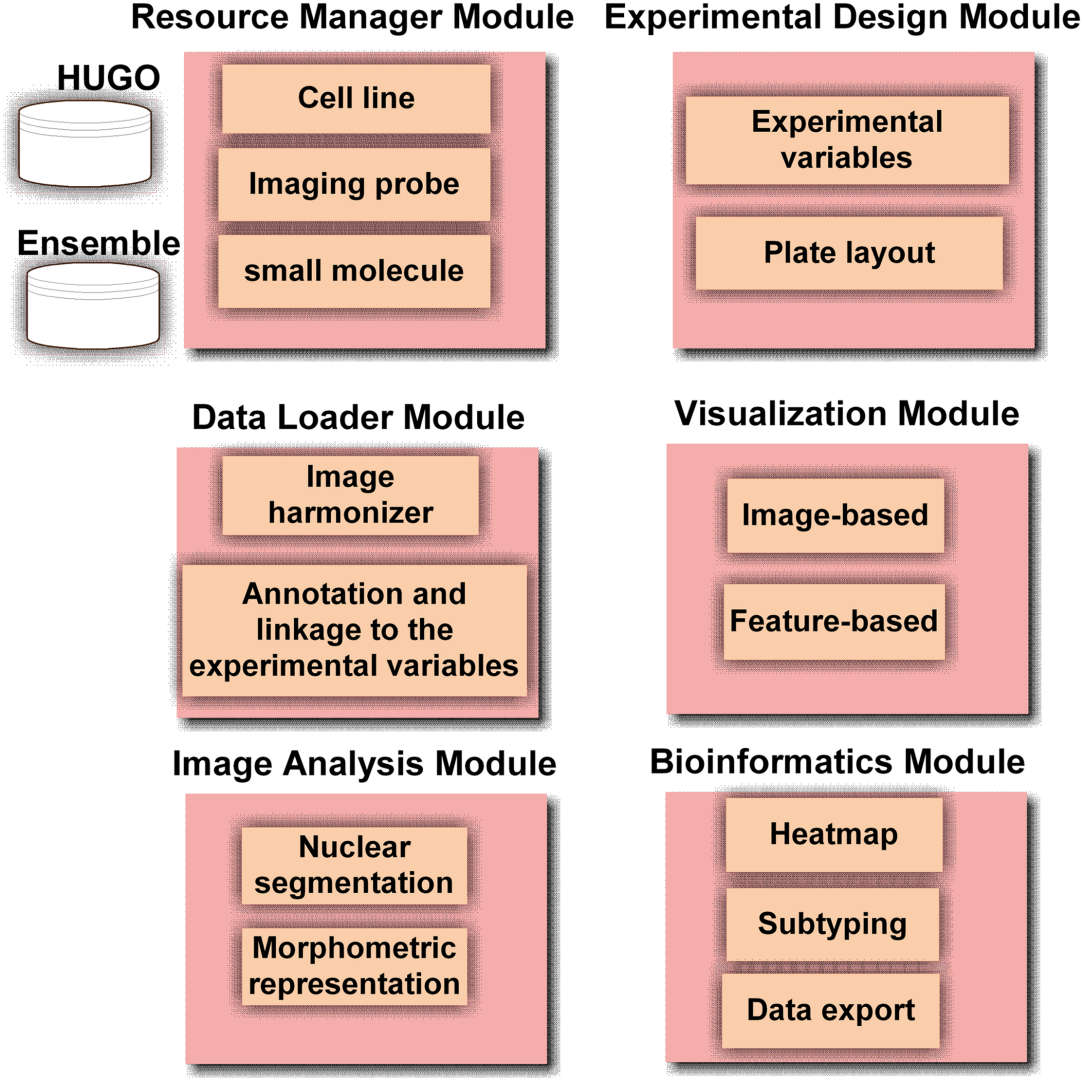
BioSig3D consists of six modules. These include resource manager, experimental design, data loader, visualization and 3D rendering, image analysis, and bioinformatics. These modules are tightly integrated with the backend database.

BioSig3D is an imaging bioinformatics system, which is designed for screening therapeutic targets and gaining new insights into biological processes, by profiling aberrant organizations. It leverages several components of Open Microscopy Environment, such as the Bioformats and OME image server (OMEIS) [14, 23]. Although 3D cell culture models provide an effective model for high content screening, they also impose a number of computational and bioinformatics challenges since the data needs to be visualized and processed in full 3D volume. The design of BioSig3D is database centric and utilizes PostgresSQL. The system has six conceptual components, listed below and shown in Fig 1, which are tightly integrated through controlled vocabularies (CVs). The CVs have been borrowed and extended from existing ontologies [24, 25]. For example, the nomenclature for specifying an antibody is coupled with the HGNC database [26], which is one of the better maintained databases for human genes. Other ontologies for antibodies are still at their infancy and HGNC is the best path for moving forward at this point. The six services are

- Resource Manager
- Experimental Design Manager
- Data Load Manager
- Visualization modules
- Image analysis modules
- Bioinformatics modules

With the exception of image analysis modules, these services are summarized in S1 Text and the companion videos. These modules are tightly coupled through a back-end PostgreSQL (PG) relational database.

#### Client interaction with BioSig3D

**Fig 2:**
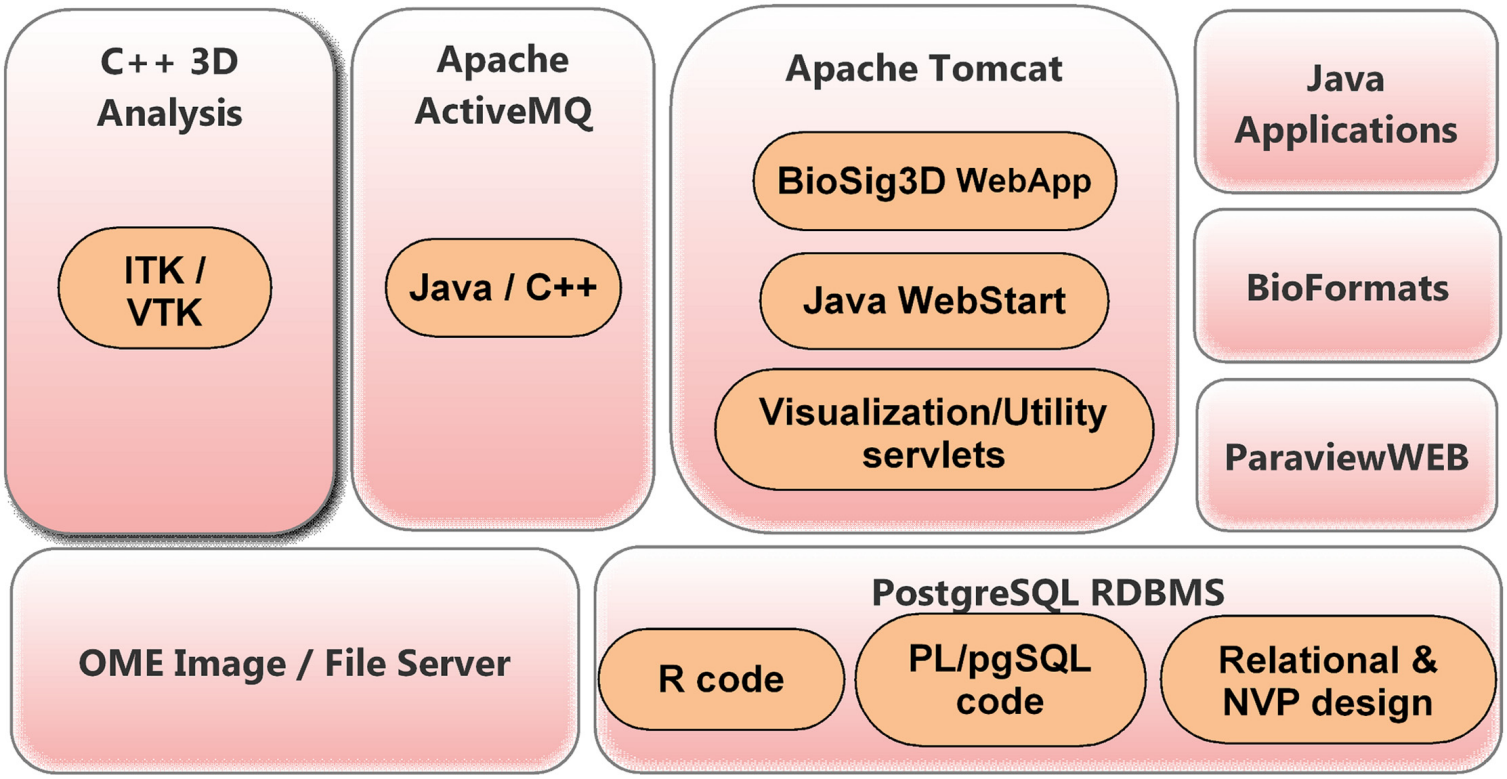
Architecture of BioSig3D is based on open source components Apache Tomcat, OME Image Server, ParaviewWeb from Kitware, and PostgreSQL database.

Computational components of BioSig3D build and extend on open source software components, shown in Fig 2. Client interaction with BioSig3D is summarized below.

Each user interaction, through the web interface, begins from the creation of studies and associated experiments. Each experiment includes experimental factors, such as cell lines, environmental perturbations, and harvest time, which are entered through the BioSig3D web application interface. A subset of a selected combination of experimental variables can then be coupled with molecular probes for imaging. Once the images have been acquired, the user can initiate the transfer of images by invoking the BioSig3D Java image importer application through Java Web Start. The image importer is integrated with the BioFormats image reading library, where image headers are read, and their attributes (*e*.*g*., image dimensions) are displayed for consistency and quality control. For example, all images, within a dataset, should have the same number of channels and the pixel size must be consistent. Once a consistent dataset is formed and selected, they are streamed from the local host to the system back-end, where both the original image files and extracted raw 5D data pixel arrays are stored in instances of the Open Microscopy Environment (OME) Image server. The rationales for this duplication are to (i) maintain the exact original data files, with their internal annotation, and (ii) decouple image analysis from various imaging formats, so that the analyses are performed only on homogenized raw image data. Next, the user links each image or group of images with their corresponding treatment conditions (*e*.*g*., experimental factors). Subsequently, the system automatically constructs thumbnails, which are displayed through the web interface.

Invocation of the image analysis jobs, on a designated dataset, is also performed through the web interface. The user can select a specific version of an algorithm (such as nuclear segmentation, or cell segmentation), configure the parameters of the algorithm, and submit this parameterized job request for execution. Since both image and bioinformatics analyses are compute-intensive, they are managed by ActiveMQ per S2 Text. The parameterized job request is posted on an Apache ActiveMQ message queue for programmatically-independent consumption. The C++ 3D ITK/VTK-integrated image analysis codes have been augmented with a C++ ActiveMQ message queue client wrapper, which processes these requested analysis job messages with specified parameters. Any number of these wrapped analysis modules can be started on any host or cluster, which allows scalability across any number of jobs in the queue. These modules are isolated from any database dependencies, and the system design facilitates the development of future computational methods, by only requiring the consumption of messages and generation of the analysis results. Once a job request message has been received, the analysis module invokes a command-line Java application to download the 5D image raw dataset. Downloaded images are first verified for data integrity and possible corruption, the image analysis task is initiated, and the task is then registered with the database. At the completion of task, the analysis code generates a single XML file per image stack that contains all of the computed features. Generated XML file (a) is validated against a predefined hierarchical XSD schema, which in addition to the required base elements, allows for any number of feature name-value pairs in a specific category (*e*.*g*., shape, intensity) to be added, and (b) contains all of the computed features in the predefined hierarchical layout. The XML Schema Definition (XSD) defines multilevel hierarchy of computed features and their relationships, and is included in S3 Text. Computed morphometric and molecular endpoints (*e*.*g*., indices) are organized at the three levels of colony organization, cell, and subcellular regions. In addition to these XML files, metafiles are also generated, which include nuclear or cell segmentation masks, exported as *ITK*.*mhd* files, and 3D visualization files that are exported as *VTK*.*vtp files*. All computed feature and metafiles are imported and stored in the database, and the relationships between computed indices, at different levels, are registered in a relational schema. Furthermore, computed numerical features are stored in name-value pair containers for increased flexibility, *i*.*e*., new features can be generated and imported dynamically. In BioSig3D, features can be (i) exported to a client’s desktop for further statistical analysis, (ii) selected for correlative analysis against experimental conditions, (iii) analyzed and visualized for heterogeneity, or (iv) visualized with heatmap in the browser. All bioinformatics analysis (e.g., heatmap, heterogeneity) are implemented in R and integrated with the database through pl/R module. In addition, computed nuclear/cell segmentation masks can be visualized without the need to download plugins, in the web browser, via an integrated VTK ParaviewWeb framework. 3D rendering is supported through Javascript and WebGL if the desktop supports a graphic card.

#### Functional views of BioSig3D

**Fig 3:**
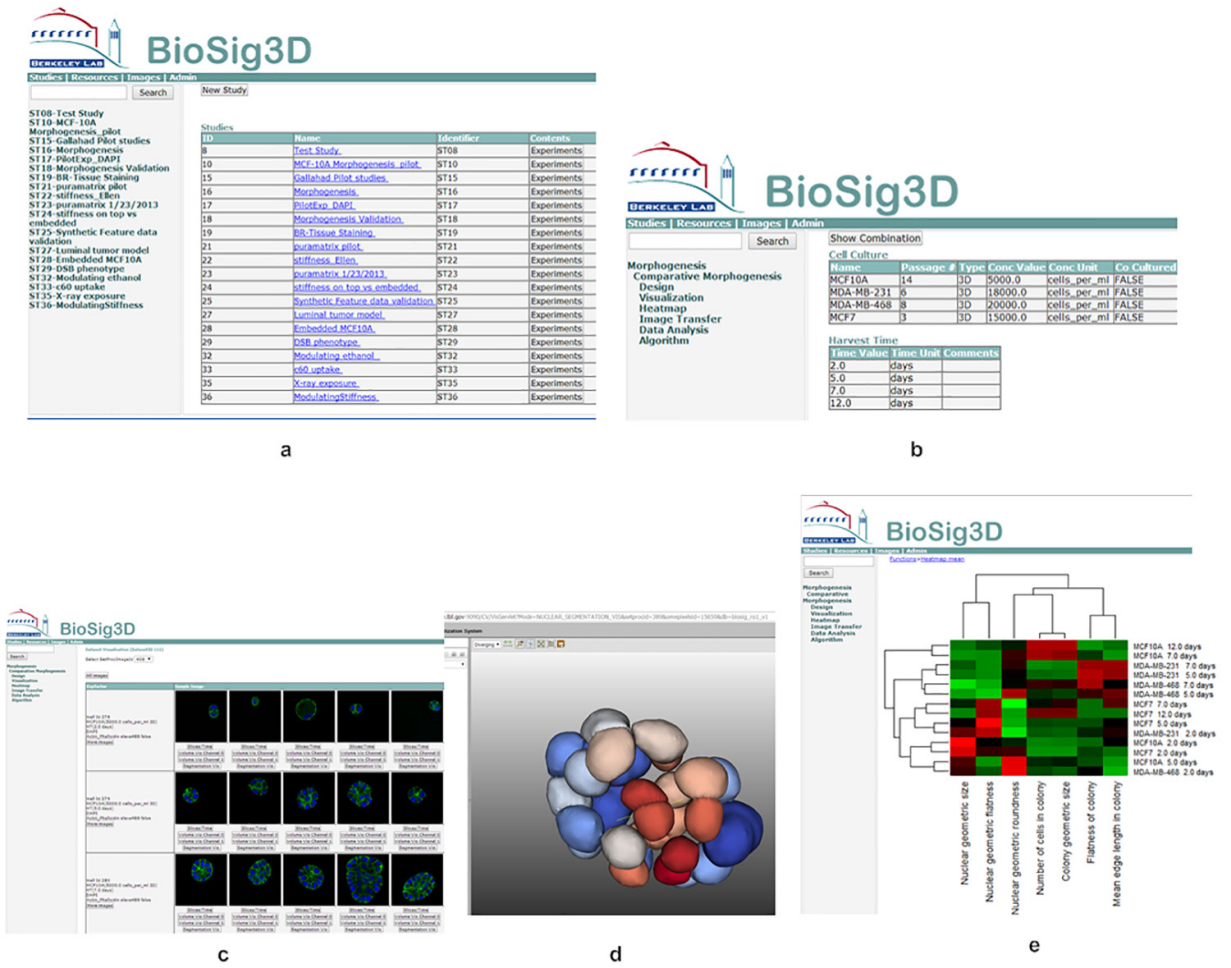
Functional view of BioSig3D includes (a) a list of studies in the system, (b) a particular study with 4 cell lines that are harvest at different time points, (c) a representative set of colonies for an experiment where only the middle slice of the 3D stack is shown, (d) Nuclei, in one of the colonies, are segmented in 3D, which can be rendered on the web for quality control, and (e) a typical bioinformatics analysis in terms of the global heatmap from computed indices.

The database features permit experimental and analytical results to be repeated, compared, and monitored. The main innovations of BioSig3D are: (i) more robust algorithms for image analyses with performance superior to the existing approaches, (ii) a new set of indices computed for aberrant phenotypes, based on colony organization, (iii) integrated visualization and bioinformatics methods, and (iv) end-to-end solutions, via Web interface, for enabling a more sophisticated approach for high-content screening. Fig 3 indicates several views of BioSig3D, where the platform:

- Quantifies nuclear morphometric indices and the corresponding molecular endpoints, on a cell-by-cell basis, using advanced image analysis algorithms, which are implemented in ITK/VTK C++ high-performance language;
- Provides quantitative measures for the analysis of aberrant colony organization; and
- Enables a variety of bioinformatics analyses, based on colony organization, nuclear morphology, and molecular endpoints;
- Enables visualization of raw and processed 3D spatial data for quality control, using a number of different modalities.

#### Image format

BioSig3D accepts data in multiple formats. These include STK (Metamorph), ZVI (Old Zeiss), LSM (new Zeiss microscopes), and OME-TIFF. There are no standards for TIFF. The VM software can be run on any operating system platform. It is recommended that the desktop should have a minimum of 4 Gigabytes of Ram. Installation software will require Apache Tomcat, PostGreSQL, and Kitware ITK/VTK library. Documentation, videos, and instruction on installation of BioSig3D are located at http://biosig.lbl.gov/BioSig3D. The video content provides a step-by-step procedure for experimental design, annotation, and processing. See S1–S6 and S22 Videos. Our goal is to extend and refine features of BioSig3D continuously, and post them on this website.

#### Timing

The timing for computational and bioinformatics analysis are shown.

- Image analysis for one stack of size 512-by-512-by-60: 4 minutes
- Visualization of rendered surfaces with JavaScript: 6 sec. WebGL is recommended for rendering, if the desktop unit has a graphic chip.
- Heat map generation: is sample size dependent with 2–3 seconds for the released dataset.

#### Controlled vocabularies

High-throughput screening cannot be decoupled from proper annotation of resources and experimental parameters, and visualization services. The present design is database-centric, which has six conceptual components that are tightly integrated through controlled vocabularies (CVs), where the CVs have been borrowed and extended from existing ontologies. The rationale for such an integrated system is to expedite and enable high-content screenings. These components are all coupled together through a back-end PostgreSQL (PG) relational database. The database features assure that experimental and analytical results can be repeated, compared, and monitored.

#### Colony organization

Colony organization is computed as a function of position for each nucleus, within the colony. BioSig3D is the first system that provides a multiparametric profiling of colony organization. Generally, it is difficult to discriminate cancer cells in a monolayer system without additional staining; however, the 3D systems present an organization that explicitly indicates an aberrant/cancer phenotype. This concept of examining colony organization, as opposed to individual cellular morphometry and distinct molecular endpoints, is a new innovative method in screening therapeutic targets. Hence, therapeutic targets can be tested in an effort to determine if they can reverse an aberrant organization, as reported earlier in [7]. Additionally, cell lines with distinct genetic defects can lead to a specific type of colony organization. For example, in the case of breast cancer cell lines, three distinct colony organizations have been identified and labelled as “mass,” “grape-like,” and “triple negative,” which correspond to luminal, generally ERBB2-positive cell lines with EGFR amplification, and triple negative [12], respectively, as shown in S4 Text.

Multiparametric colony organization is expressed as the (i) global shape of the 3D colony (e.g., roundness, flatness, elongation), and (ii) local features of the 3D colony, which is computed as a function of the position of each cell in the colony, with respect to the convex hull of the colony. From these computed indices, meta-features are also computed to reflect other important attributes, such as lumen formation in normal mammary structure. Lumen formation, in HMEC, involves a complex series of biological processes that lead to polarized cellular organization. However, polarity requires additional antibody staining (e.g., ZO1, beta4-integrin), which increases the cost of sample preparation, HCS, and computational assay. An approximate index for representing lumen formation is to quantify the empty space inside the colony, which serves as an initial low cost screen for profiling colony organization. This is a more rational approach since the absence of an empty space, within the colony, is likely due to a mass formation with unpolarized cells. A complete list of computed indices from colony organizational features is included in S4 Text. These indices include those that are derived from spatial geometrical conformation and those that are computed from graph-based representation of each colony. In summary, quantitative profiling of colony organization is an important component for high-content screening. It will reduce the cost of labeling additional molecular endpoints for imaging and provides an explicit readout.

#### Visualization and bioinformatics analysis

Components of BioSig3D for visualization of volume and segmented 3D surfaces are presented in S1 Text, which also provides several features for Bioinformatics analysis: (i) cell-by-cell morphometric analysis, (ii) colony organization, (iii) heterogeneity analysis, and (iv) correlative analysis between nuclei/cell-based measurement and organizational features. These features can then be displayed as heat maps or false discovery rate (FDR)-corrected correlative analysis. Among these features, heterogeneity analysis is an important facet of colony organization, which relies on subtyping based consensus clustering [27]. BioSig3D can evaluate morphometric indices individually, or in combination with each other. Subtyping is based on the classical K-Means algorithm, with the Euclidean distance. The resampling rate and number of iterations are set at 0.8 and 200, respectively, and the clustering results for 2 to 5 subtypes are shown as similarity matrices to the end user. Subsequently, the user can select the number of subtypes for specified indices One can repeat consensus clustering on a randomly selected subset of data (e.g., for example with sampling rate = 0.8) for a number of times (e.g., typically 100 iterations) to visualize bar charts, corresponding to subtypes, with a mean and standard error of colony distribution across different subtypes. In addition, the client can visualize populations of colonies that correspond to each subtype and a representative model of each subtype. This is often referred to as content-based retrieval from spatial data.

#### BioSig3D packaging

Present release of BioSig3D is through the VMWare ESXi, which is the industry-leading virtual machine server platform. ESXi is a type 1 hypervisor [28] meaning that it runs as the operating system on the host machine. Type 1 hypervisors enable significantly better performance and control of resources for multiple hosted virtual machines. Also in contrast to VirtualBox, a type 2 hypervisor, the VMWare tools allow for very fine-grained resource allocation and monitoring across hosted virtual machines. Additional videos and documentations are included at the BioSig website.

## Results and Discussion

BioSig3D has been tested and validated for high content screening (HCS) of 3D cell culture models. It allows for the design of an HCS experiment, sharing the data with other users, and processing large amount of 3D data for bioinformatics analysis. Presently, BioSig3D provides morphometric profiling by quantifying cellular and colony organization indices, which will enhance interpretation of biological processes and integration with genome-wide molecular data [12]. In this section, the performance of the nuclear segmentation, presented earlier in [11] is summarized, followed by two case studies.

### Nuclear segmentation modules

One of the key computational components is nuclear segmentation from the full 3D volume stacks [11], which was published earlier. Complexities arise from variations in shape and size of each nucleus and the fact that adjacent nuclei can overlap, as a result of fixation, or lack of resolution along the z-axis, which is well documented in prior literature [29, 30]. Nuclear segmentation (i) provides the context for quantitative analysis of other molecular endpoints, and (ii) enables quantitative analysis of the aberrant colony organization. Image analysis methods have been evaluated with both synthetic and real data. Synthetic data aims to emulate images with a variety of cellular configurations that are corrupted by different types of noise, where the signal-to-noise ratio of 9dB was used for detailed profiling [11]. With respect to real data, BioSig3D couples experimental design and computational methods for algorithmic validation. Accordingly, the experimental design includes four mammary cell lines of epithelial origin, with diverse genetic backgrounds. These are (a) MCF7, which is progesterone positive, estrogen positive, and ERBB2 negative; (b) MDA-MB-468, which is ERBB2 negative with EGFR amplification; (c) MDA-MB-231, which is progesterone negative, estrogen negative, and ERBB2 negative; and (d) MCF10A, which is nontransformed and recapitulate lumen formation. Subsequently, colony formations were harvested in days 2, 5, 7, and 12. Each cell line contributes uniquely to 3D colony organization for validating higher order bioinformatics analysis. In every imaging bioinformatics system, nuclear segmentation is the key computational engine for high order bioinformatics analysis. BioSig3D uses a technique, known as curvature based partitioning (CBP) [11], which has a superior performance over watershed methods or its variant of marker-based watershed, gradient flow tracking (GFT) [31], and iterative voting [29]. Performances in terms of F-measure, derived from precision and recall, are shown in Tables 2 and 3.

**Table 2:** F-measure is computed for 50 randomly generated synthetic colonies with added noise of different categories. CBP consistently outperforms competing approaches.

F-measure	Gaussian Noise	Shot Noise	Speckle Noise	Salt and Pepper Noise
Curvature-based partitioning (CBP)	1.00	1.00	1.00	1.00
Iterative Voting	0.97	0.95	0.99	0.88
Gradient flow tracking (GFT)	0.69	0.61	0.60	0.75

**Table 3:** CBP outperforms other methods in nuclear segmentation.

	Watershed	Marker-based Watershed	GFT	Iterative Voting	CBP
F-measure	0.71	0.80	0.88	0.87	0.97

### Case studies

Two case studies that involve baseline analysis of colony organizations are presented here. In both cases, experiments are designed in triplicates, and samples are imaged with confocal microscopy in full 3D. Because of the slow growth rate properties of the 3D colonies, in terms of days, BioSig3D does not support dynamic imaging, which also requires specialized instrumentation and imaging probes. Instead, dynamic imaging is limited to population studies at a fixed harvest time of independent samples.

**Fig 4:**
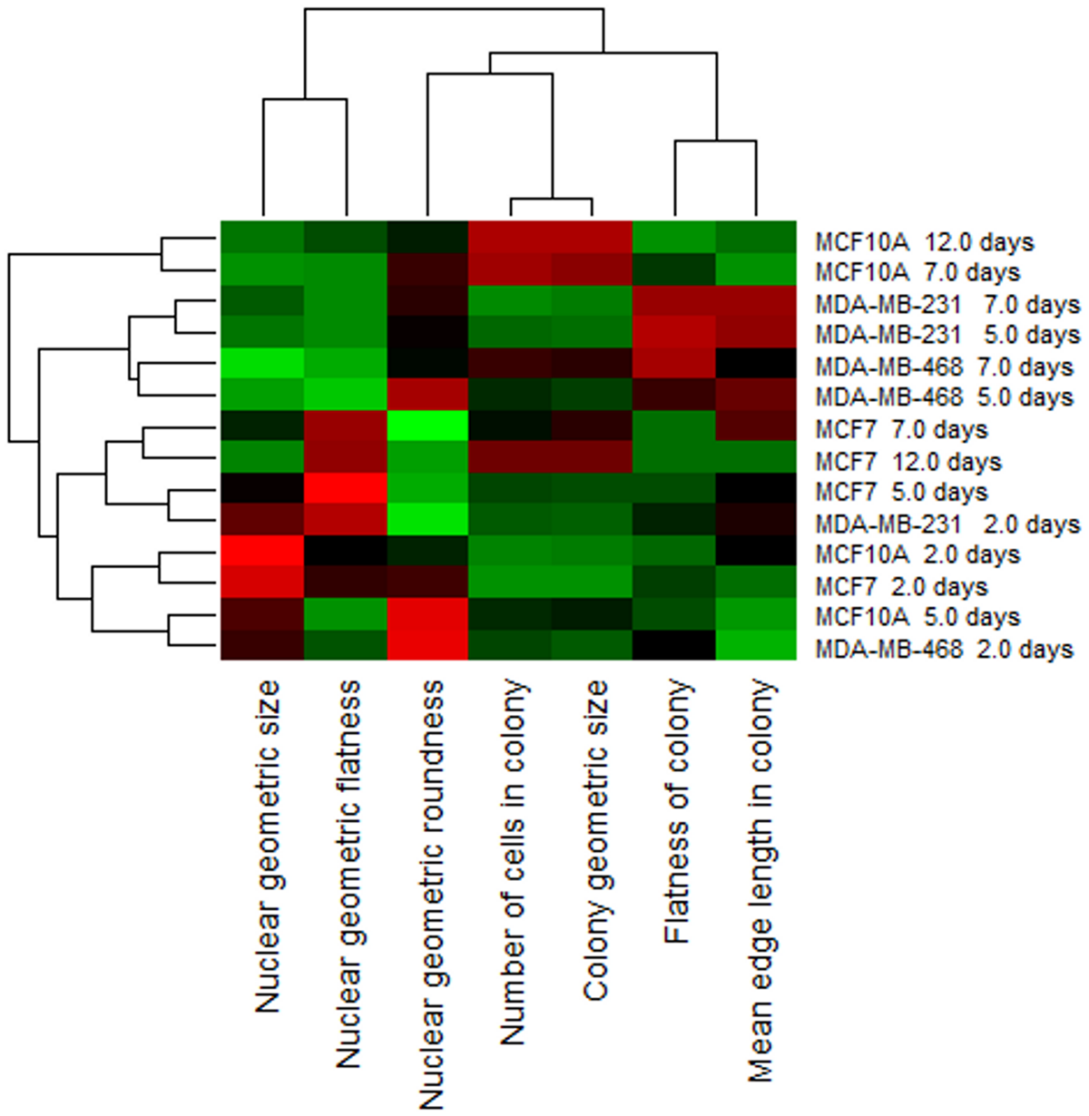
A heat map summarizing a dataset corresponding to the morphogenesis of 4 different human mammary cell (HMEC) lines with diverse genetic background.

In the first study, we examined the growth properties of the four human mammary cell lines that were used for validation, in terms of nuclear shape features and colony organization [11], with the heatmap results shown in Fig 4. In this study, with the exception of MDA-MB-231 and MDA-MB-468, all samples were fixed on days 2, 5, 7, and 12. Profilings of MDA-MB-231 and MDA-MB-468 for day 12 were excluded because they grew too fast and exceeded the maximum allowable sample thickness of our imaging instrument. For each experimental conditions (e.g., cell line, harvest time), between 9 and 12 samples were imaged. The findings are summarized below, and some of these results correlate with the published literature [32].

- At the nuclear morphometric level, two of the cell lines with noninvasive properties (e.g., MCF10A, MCF7) reveal larger nuclei in the early stages of colony formation; however, the size of the nuclei shrinks as the size of the colony expands. In contrast, for invasive cell lines (e.g., MDA-MB-231, MDA-MB-468), nuclear size does not shrink as a function of morphogenesis and colony development.
- At the colony organization level,
  - In the later stages of colony formation, colony shapes are round and elongated for noninvasive and invasive cell lines, respectively;
  - The average distance between neighboring cells is lower in the early time points, as compared with the later time points of colony formation. This property is invariant of the cell lines;
  - The premalignant line, MCF10A, has the capacity for lumen formation, while other cell lines do not; and
  - MCF7 has a slower proliferation property than MCF10A with individual nuclei being more flat (e.g., more disk shape) than round.

**Fig 5:**
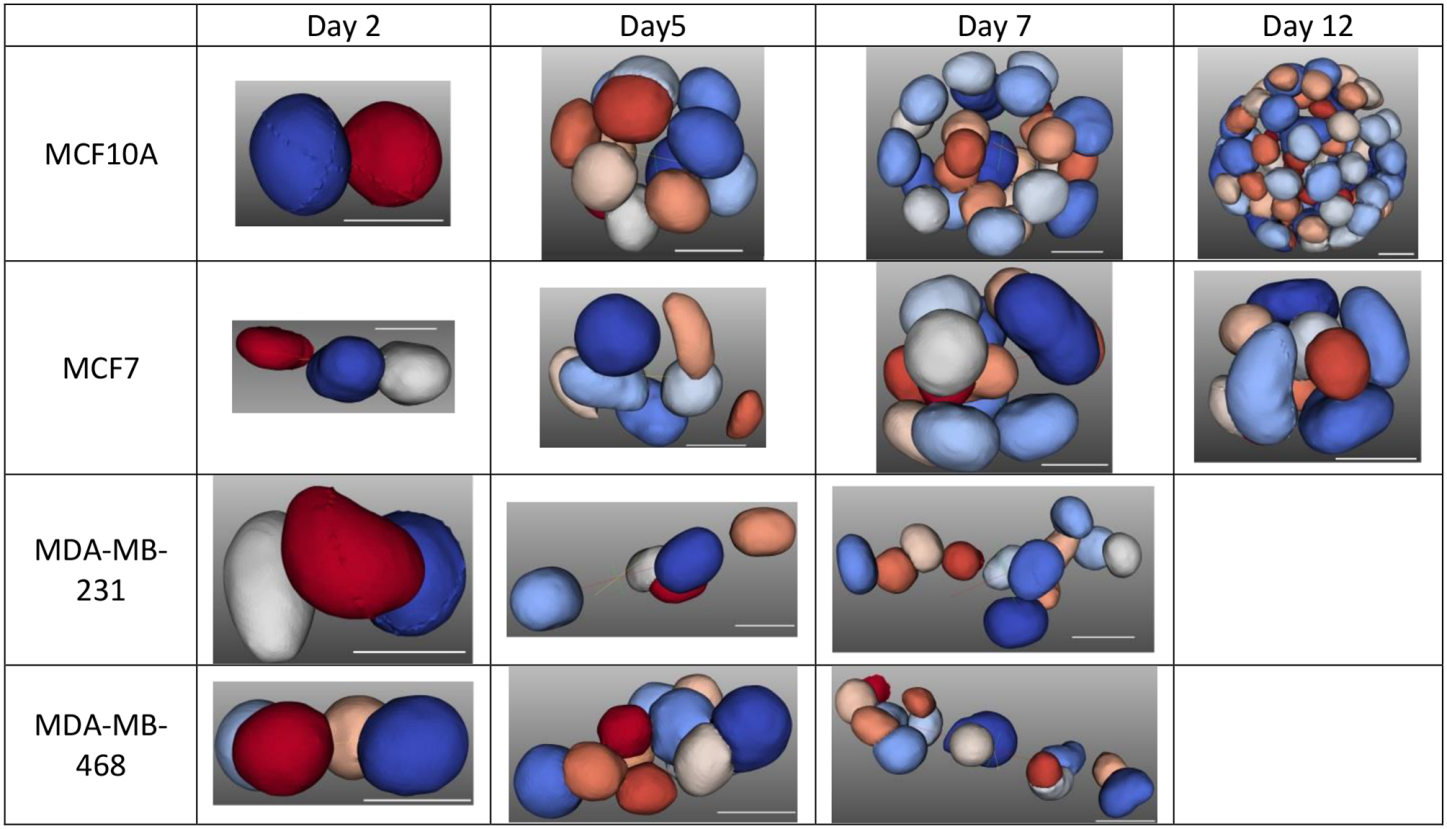
Representatives of colony formations, for the heatmap of Fig 4, for each cell line that is fixed one a specific date. The scale bar is 10 microns.

**Fig 6:**
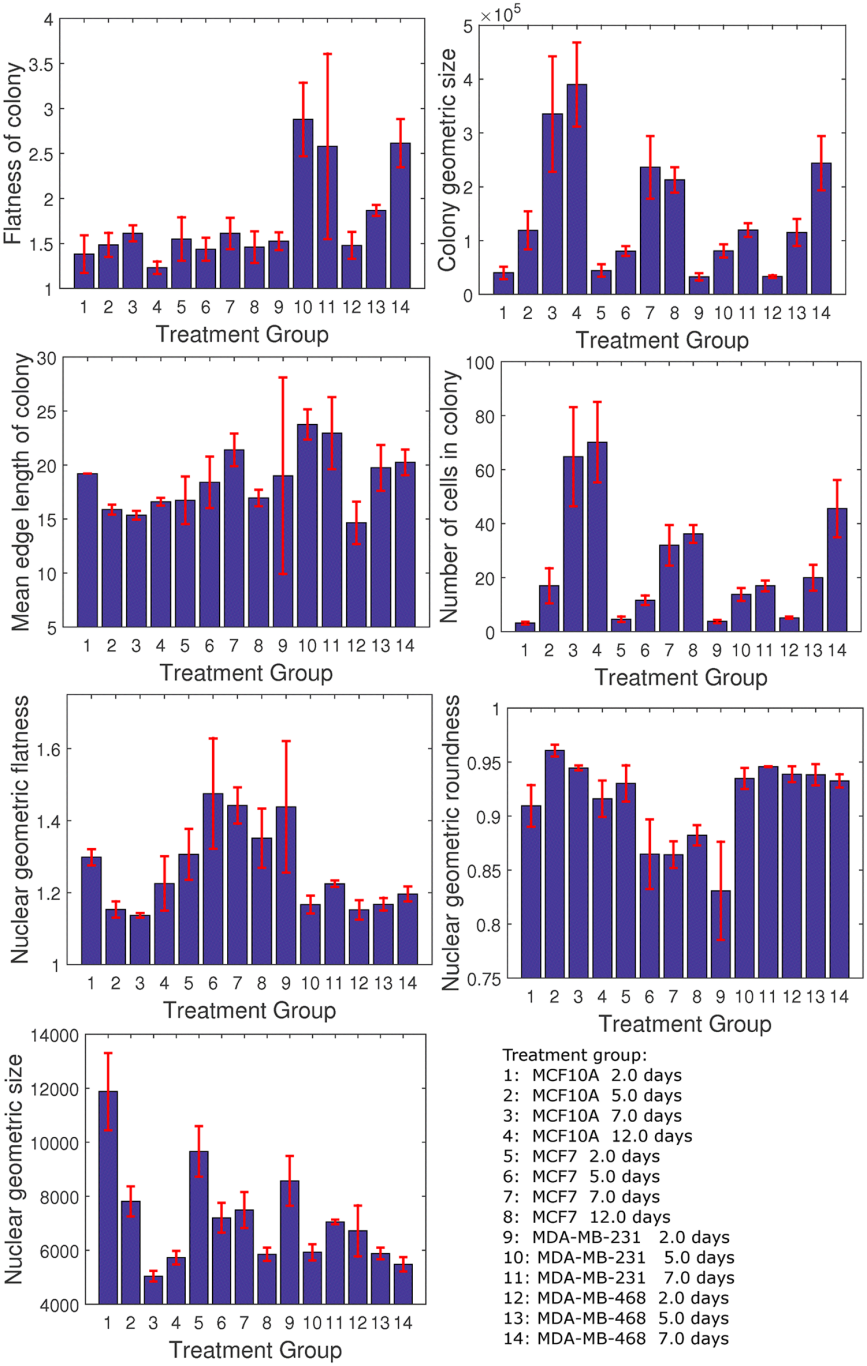
Population analysis for the heatmap of Fig 4 indicates stability of computed indices. In some cases, flatness of colony is referred to as “Indian file pattern” in clinical pathology.

Fig 5 shows a representative set of colony formation for each of the cell lines, for the heat map shown in Fig 4. Some of these observations (e.g., lumen formation in MCF10A) are consistent with the published literature [7, 13]. Furthermore, they reveal that (i) the development of the spherical or planar colony formation dictates nuclear size, independent of the distances between nuclei within the colony, (ii) aberrant spherical colony formation leads to nuclei that are more flat than round, and (iii) in some cases, as shown in Fig 6, computed indices have a large variation, requiring either a larger sample size or heterogeneity analysis.

**Fig 7:**
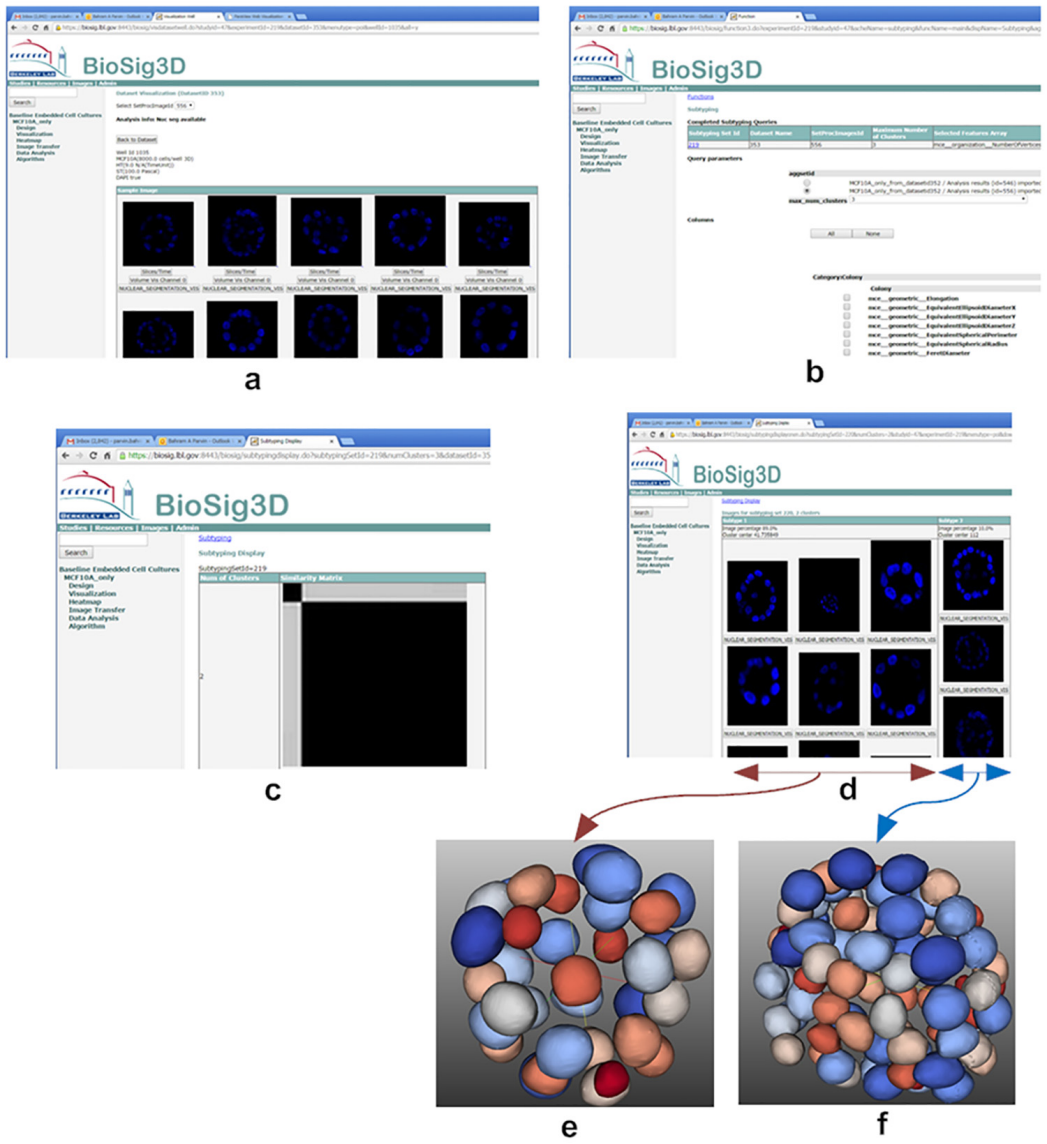
Colony formation is heterogeneous under normal growth condition for MCF10A (a non-transformed HMEC cell line). (a) a representative of colonies shown as thumbnails with the middle section of the 3D volume displayed, (b) client can select from a list of computed indices (e.g., colony size) for subtyping, (c) two subtypes, shown as a similarity matrix with two dominant blocks, are inferred for the computed index corresponding to the number of cells per colony, (d) the client queries for a representative population to view each subtype in its own column, and (e-f) rendered representative colonies for each of the subtype clearly indicates that one subtype has more cells than the other.

In the second study, we examined heterogeneity in colony formation for MCF10A, using embedded 3D cell culture model. Samples were imaged in 3D, and 59 colonies were uploaded into BioSig3D. In each colony, nuclei were segmented, and colony indices were computed. Heterogeneity, in colony formation, was then quantified by means of subtyping using consensus clustering [27], which has been integrated into BioSig3D. The user can select the maximum number of clusters, examine computed similarity matrices, and view the corresponding population for each subtype. This experiment revealed that there are two subtypes of MCF10A with a differential proliferation rates (e.g., number of cells in each colony). In these two subtypes, the mean colony sizes were 42 and 112 cells, respectively. The process of computing and displaying subtypes are shown in Fig 7, where Fig 7c indicates two subtypes in the similarity matrix, as a result of consensus clustering (e.g., two dominant blocks), and Fig 7d tabulates and orders colonies that belong to each subtype. Fig 7e and 7f show a representative of colony formation for each of the subtypes. The experiment suggests that the MCF10A cell lines do not have a homogeneous proliferation rate in 3D. The concept of querying colony organization as a function of computed indices is often referred to as content-based retrieval [20]. Typically, in the literature, the results are shown for representative growth properties, but it is important to note the baseline heterogeneity may be an important factor in designing sensitive assays. Several publications have also indicated that phenotypic heterogeneity is not an exception [33, 34]. Future experiments may include sub-culturing from highly proliferating colonies to examine whether the original population can be reconstructed, or a highly proliferative derived cell lines can be acquired.

## Conclusions

High content screening of colony organization from 3D cell culture models are highly informative, but are intrinsically complex. These complexities originate from the multifactorial experimental design (e.g., cell lines, therapeutic targets, concentration, harvest time), which need to be associated computed indices from volumetric images. BioSig3D provides a solution for (i) annotation of experimental variables through control vocabulary, (ii) linking 3D imaged samples to experimental variables for visualization and quality control, (iii) computing morphometric properties of the 3D cell culture models that are imaged in 3D, and (iv) providing integrated tools for bioinformatics analysis. The latter includes both global (e.g., heatmap) and heterogeneity (e.g., subtyping through consensus clustering) analysis. The system has been applied to two baseline studies that include (i) growth properties of human mammary cell lines with diverse genetic properties, and (ii) heterogeneity analysis of a non-transformed cell line. The first experiment was cultured using the “on-top” method and provides a reference for the 3D colony organization for each of the cell lines. The second experiment was cultured using the “embedded” method to conclude that the commonly used pre-malignant MCF10A cell line has a heterogeneous growth property with two distinct subtypes. These baseline experiments provide the researchers a template for how to use BioSig3D and to extract additional information that is typically ignored or hidden (e.g., colony organization, heterogeneity). The long-term benefit is a more effective screening of therapeutic targets, while reducing *in vivo* testing. As a result, the cost of screening therapeutic targets is decreased and the screening process time is shortened. Furthermore, biological processes can be better studied in systems that more faithfully recapitulate realistic models. BioSig3D is going through incremental evolution and new versions will be released to the community.

## Supporting Information

S1 Text BioSig3D Services. (DOCX)

S2 Text Integration of image analysis and visualization modules. (DOCX)

S3 Text Schema definition for computed indices. (PDF)

S4 Text Colony organization and computed indices. (DOCX)

S1 Video (BioSig3D: Resource Manager Video): A video presenting the use and application of resource manager. (MP4)

S2 Video (BioSig3D: Experimental Design Video) A video presenting the process of experimental design in BioSig3D. (MP4)

S3 Video (BioSig3D: Plate Layout Video) A video presenting how to do plate layout for high content screening. (MP4)

S4 Video (BioSig3D Uploading Images Video) A video presenting how to upload images into BioSig3D for visualization of processing. (MP4)

S5 Video (BioSig3D: Image linking video with the experimental variables) A video presenting how to use the java app to link images with experimental factors. (MP4)

S6 Video (Biosig3D: Visualization and bioinformatics analysis) A video presenting visualization and bioinformatics analysis in BioSig3D. (MP4)

S7 Video 3D stack for a representative of MCF10A at day 2 with scale (first example). (AVI)

S8 Video 3D stack for a representative of MCF10A at day 2 with scale (second example). (AVI)

S9 Video 3D stack for a representative of MCF10A at day 5 with scale. (AVI)

S10 Video 3D stack for a representative of MCF10A at day 7 with scale. (AVI)

S11 Video 3D stack for a representative of MCF10A at day 12 with scale. (AVI)

S12 Video 3D stack for a representative of MCF7at day 2 with scale. (AVI)

S13 Video 3D stack for a representative of MCF7 at day 5 with scale. (AVI)

S14 Video 3D stack for a representative of MCF7 at day 7 with scale. (AVI)

S15 Video 3D stack for a representative of MCF7 at day 12 with scale. (AVI)

S16 Video 3D stack for a representative of MDA-MB-231at day 2 with scale. (AVI)

S17 Video 3D stack for a representative of MDA-MB-231at day 5 with scale. (AVI)

S18 Video 3D stack for a representative of MDA-MB-231at day 7 with scale. (AVI)

S19 Video 3D stack for a representative of MDA-MB-468 at day 2 with scale. (AVI)

S20 Video 3D stack for a representative of MDA-MB-468 at day 5 with scale. (AVI)

S21 Video 3D stack for a representative of MDA-MB-468 at day 7 with scale. (AVI)

S22 Video BioSig3D overview. (MP4)
