# Supplementary material for: BioSig3D: High Content Screening of Three-Dimensional Cell Culture Models
Source: PLoS One. 2016 Mar 15;11(3):e0148379. doi: 10.1371/journal.pone.0148379 (PMC4792475; doi:10.1371/journal.pone.0148379)
Supplement: S1 Text — (DOCX) [file pone.0148379.s001.docx]

**S1 Text: BioSig3D services**

The rationale for such an integrated system is to expedite and enable high-content screenings. These components are all coupled together through a backend PostgreSQL (PG) relational database. The database features permit experimental and analytical results to be repeated, compared, and monitored. Since both image analysis and visualization are compute-intensive, they are managed by ActiveMQ per Supplementary 3.

The Resource Manager (RM) maintains an inventory of cell lines, reagents, antibodies, and small molecule inhibitors. Imaging probes are labeled as counterstains, chemical stains, or antibodies, where the antibodies use control-vocabularies from the HUGO gene nomenclature. Every resource follows its own template to track associated ontologies and requires a minimal set of laboratory annotations. For example, a cell line will have attributes associated with its origin and growth conditions.

The Experimental Design Manager (EDM) interacts with the RM to design an experiment utilizing a series of templates and CVs. It is well understood that the experimental designs may consist of many atomic steps with endpoints of considerable complexities, e.g., evaluating components of a growth media on a colony formation. The EDM design, in BioSig3D, is motivated by three distinct goals: (i) imposing a minimal set of requirements for annotations, (ii) focusing on high-content screening as opposed to assay development, and (iii) the inclusion of multiple cell lines (e.g., a panel of breast cancer cell lines with different molecular aberrations) for screening. The net results are a reduction in system complexity, an improvement in user interactions, and a more effective screening of therapeutic targets.

The Data Load Manager (DLM) supports three functionalities: (i) uploading data in a variety of input formats into the image server, (ii) linking data with associated experimental factors and individual wells in a multiwell plate format, and (iii) providing utilities for downloading images and computed representations. One of the main barriers to interacting with images involves the variety of formats that imaging instruments use to export their data. This issue has already been addressed within the Open Microscopy Environment Remote Objects (OMERO) community [[1](#_ENREF_1)] , where their solution has been the introduction of Bioformat and an image server for homogenizing image representation. Bioformats and OME image servers have been integrated into the BioSig3D for compatibility and exchange of image-based data [[2](#_ENREF_2)].

Visualization modules support three distinct services: (i) a thumbnail viewer of an image stack, (ii) volume visualization, and (iii) surface rendering of processed samples. The thumbnail viewer shows one slice from the middle of a 3D stack, hyperlinked to all of the slices within the image stack stored in the OME image server. Volume and surface visualization leverage Kitware’s ParaView application, through the ParaViewWeb extension, running in an Apache Tomcat instance. Installed on a server host, ParaViewWeb provides an interactive interface to visualize and analyze scientific data sets via a communication bridge. ParaViewWeb offers multiple options for client-server communication methods, including JavaScript calls and WebGL. While WebGL provides the highest level of interactive performance, JavaScript provides greater compatibility with different browsers and video cards. The Tomcat web server hosts all the web applications. For example, the Vis-servlet interacts with the OMEIS to generate MHD files that are needed by ParaviewWeb for volume visualization. The MHD files are automatically generated when the data is processed and stored in OMEIS. The image analysis software independently generates the VTP files for quality control of the segmentation results, which are also stored on OMEIS repository. For volume visualization, a series of JPEG files are extracted from the OME image-server to construct an intermediate file in Kitware’s MHD format. For surface visualization, segmented surfaces are generated and stored in Kitware’s VTP format, which is also stored in the OME image-server. ParaviewWeb can then render various views form these intermediaries. The S1 Fig shows interactions between the web client, visualization modules, and OME image server.


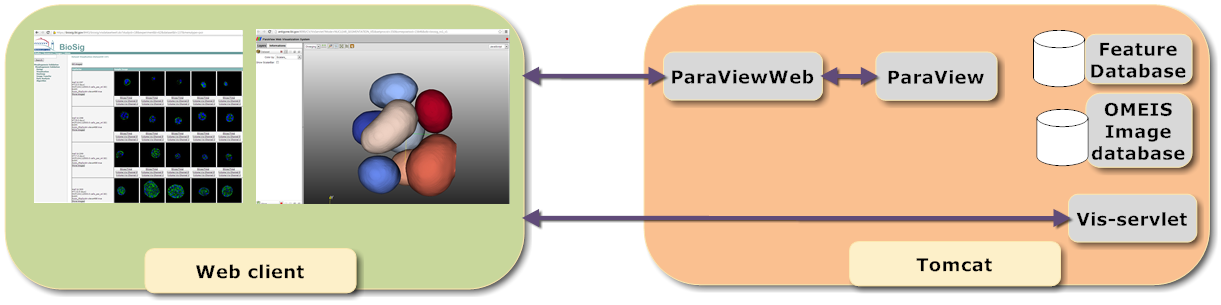


Fig A in S1 Text: Interaction between the Web client and Visualization modules

1. Goldberg, I., C. Allan, J.M. Burel, A. Creager, H. Falconi, H. Hochheiser, J. Johnston, J. Mellen, P. Sorger, and J. Swedlow, *The open microscopy environment (OME) data model and xml files: open tools for informatics and quantitative analysis in biological images.* Cell Science 2005. **6**.

2. Swedlow, J., S. Lewise, and I. Goldberg, *Modeling data across labs.* Nature Cell Biology, 2006. **8**: p. 1190-1194.
