# Supplementary material for: BioSig3D: High Content Screening of Three-Dimensional Cell Culture Models
Source: PLoS One. 2016 Mar 15;11(3):e0148379. doi: 10.1371/journal.pone.0148379 (PMC4792475; doi:10.1371/journal.pone.0148379)
Supplement: S2 Text — (DOCX) [file pone.0148379.s002.docx]

**S2 Text: Integration of image analysis and visualization modules**

Image analysis and visualization methods are directly launched through the web, and the software architecture allows multiple datasets to be run concurrently. The software architecture is designed to be extensible for integrating continuously evolving analysis software that will be integrated into the system.

In the case of image analysis, the submission is initiated with a target set of images, a specific version of the algorithm, and associated parameters that are placed in a message queue.

BioSig3D uses Apache ActiveMQ, a robust message queue implementation, which supports numerous language bindings. A thin layer of C++ code is used as a wrapper for the C++ analysis module and serves to reduce architectural demands on the computational modules.  When a module is activated, this wrapper is responsible for relaying a specific computational method and version to a system-designated queue.  The wrapper is then responsible for monitoring the message queue and upon finding a compatible message (e.g., configuration of module name and version supported by that particular analysis module) consumes the message. The message also includes target set id and all user-set parameters. The wrapper homogenizes a representation based on ICS format and downloads the data into a local storage for image analysis. When analysis has been completed, a morphometric representation is generated, in XML, and the wrapper imports the feature data to the PostgreSQL instance.  The completion of the analysis run is registered by the analytical services, where the end user can immediately view segmented results for quality control.
