# Supplementary material for: BioSig3D: High Content Screening of Three-Dimensional Cell Culture Models
Source: PLoS One. 2016 Mar 15;11(3):e0148379. doi: 10.1371/journal.pone.0148379 (PMC4792475; doi:10.1371/journal.pone.0148379)
Supplement: S3 Text — (PDF) [file pone.0148379.s003.pdf]

```
<?xml version="1.0" encoding="UTF-8" standalone="no"?>
<!--W3C Schema generated by XMLSpy v2006 rel. 3 sp2 (http://www.altova.com)-->
<xs:schema xmlns:xs="http://www.w3.org/2001/XMLSchema" elementFormDefault="qualified">
        <xs:import namespace="http://www.w3.org/XML/1998/namespace"/>
        <xs:element name="ImageProcessing">
                <xs:annotation>
                </xs:annotation>
                <xs:complexType>
                        <xs:sequence>
                                <xs:choice>
                                        <xs:element ref="FileBasedAccessMode"/>
                                        <xs:element ref="OMEBasedAccessMode"/>
                                </xs:choice>
                                <xs:element ref="SoftwareParams_NVP"/>
                                <xs:element ref="ImageFeatures"/>
                        </xs:sequence>
                        <xs:attribute name="number_of_channels" type="xs:integer" use="
required"/>
                        <xs:attribute name="date" type="xs:dateTime" use="optional"/>
                        <xs:attribute name="hostname" type="xs:string" use="optional"/>
                        <xs:attribute name="software" type="xs:string"/>
                        <xs:attribute name="version" type="xs:string" use="required"/>
                        <xs:attribute name="is3D" type="xs:boolean" use="required"/>
                </xs:complexType>
        </xs:element>
        <xs:element name="FileBasedAccessMode">
                <xs:complexType>
                        <xs:sequence>
                                <xs:element ref="ImageFileSet"/>
                        </xs:sequence>
                </xs:complexType>
        </xs:element>
        <xs:element name="OMEBasedAccessMode">
                <xs:complexType>
                        <xs:attribute name="OMEServerURL" type="xs:anyURI" use="require
d"/>
                        <xs:attribute name="OMEPixelsID" type="xs:integer" use="require
d"/>
                </xs:complexType>
        </xs:element>
        <xs:element name="NVProp">
                <xs:complexType>
                        <xs:attribute name="name" type="xs:string" use="required"/>
                        <xs:attribute name="ordinal" type="xs:anySimpleType"/>
                        <xs:attribute name="valuetype" type="xs:NMTOKEN" use="required"
>
                                <xs:annotation>
                                        <xs:documentation>need</xs:documentation>
                                </xs:annotation>
                        </xs:attribute>
                        <xs:attribute name="value" type="xs:anySimpleType" use="require
d"/>
                </xs:complexType>
        </xs:element>
        <xs:element name="SoftwareParams_NVP">
                <xs:complexType>
                        <xs:sequence>
                                <xs:element ref="ProcessingParameterSet" minOccurs="0"
maxOccurs="unbounded"/>
                                <xs:element ref="NVProp" minOccurs="0" maxOccurs="unbou
nded"/>
                        </xs:sequence>
                </xs:complexType>
```

```
        </xs:element>
        <xs:element name="Composition_NVP">
                <xs:complexType>
                        <xs:sequence>
                                <xs:element ref="NVProp" minOccurs="0" maxOccurs="unbou
nded"/>
                        </xs:sequence>
                </xs:complexType>
        </xs:element>
        <xs:element name="Geometric_NVP">
                <xs:complexType>
                        <xs:sequence>
                                <xs:element ref="NVProp" minOccurs="0" maxOccurs="unbou
nded"/>
                        </xs:sequence>
                </xs:complexType>
        </xs:element>
        <xs:element name="Intensity_NVP">
                <xs:complexType>
                        <xs:sequence>
                                <xs:element ref="NVProp" minOccurs="0" maxOccurs="unbou
nded"/>
                        </xs:sequence>
                </xs:complexType>
        </xs:element>
        <xs:element name="Organization_NVP">
                <xs:complexType>
                        <xs:sequence>
                                <xs:element ref="NVProp" minOccurs="0" maxOccurs="unbou
nded"/>
                        </xs:sequence>
                </xs:complexType>
        </xs:element>
        <xs:element name="MeanTexture_NVP">
                <xs:complexType>
                        <xs:sequence>
                                <xs:element ref="NVProp" minOccurs="0" maxOccurs="unbou
nded"/>
                        </xs:sequence>
                </xs:complexType>
        </xs:element>
        <xs:element name="MeanCompartment_NVP">
                <xs:complexType>
                        <xs:sequence>
                                <xs:element ref="NVProp" minOccurs="0" maxOccurs="unbou
nded"/>
                        </xs:sequence>
                </xs:complexType>
        </xs:element>
        <xs:element name="Position_NVP">
                <xs:complexType>
                        <xs:sequence>
                                <xs:element ref="NVProp" minOccurs="0" maxOccurs="unbou
nded"/>
                        </xs:sequence>
                </xs:complexType>
        </xs:element>
        <xs:element name="ImageFeatures">
                <xs:complexType>
                        <xs:sequence>
                                <xs:element ref="ImageFeature" minOccurs="0" maxOccurs=
"unbounded"/>
                                <xs:element ref="MCE" maxOccurs="unbounded"/>
```

```
                                <xs:element ref="ECM" minOccurs="0" maxOccurs="unbounde
d"/>
                                <xs:element ref="ImageContour" minOccurs="0" maxOccurs=
"unbounded"/>
                                <xs:element ref="TextureParameters" minOccurs="0"/>
                        </xs:sequence>
                </xs:complexType>
        </xs:element>
        <xs:element name="ImageFeature">
                <xs:complexType>
                        <xs:all>
                                <xs:element ref="Composition_NVP" minOccurs="0"/>
                        </xs:all>
                        <xs:attribute name="channelindex" type="xs:integer" use="requir
ed"/>
                        <xs:attribute name="meanBackgroundIntensity" type="xs:float" us
e="required"/>
                        <xs:attribute name="meanForegroundIntensity" type="xs:float" us
e="required"/>
                        <xs:attribute name="stdDevBackgroundIntensity" type="xs:float"
use="required"/>
                        <xs:attribute name="stdDevForegroundIntensity" type="xs:float"
use="required"/>
                        <xs:attribute name="srcCounterStainChannelIndex" type="xs:integ
er" use="required"/>
                </xs:complexType>
        </xs:element>
        <xs:element name="MCE">
                <xs:complexType>
                        <xs:sequence>
                                <xs:element ref="Intensity_NVP" minOccurs="0"/>
                                <xs:element ref="Geometric_NVP" minOccurs="0"/>
                                <xs:element ref="Organization_NVP" minOccurs="0"/>
                                <xs:element ref="Texture" minOccurs="0"/>
                                <xs:element ref="Cell" minOccurs="0" maxOccurs="unbound
ed"/>
                                <xs:element ref="CellAdjList" minOccurs="0"/>
                        </xs:sequence>
                        <xs:attribute name="id" type="xs:integer" use="required"/>
                        <xs:attribute name="isColony" type="xs:boolean" use="required"/
>
                        <xs:attribute name="contour_startx" type="xs:integer" use="requ
ired"/>
                        <xs:attribute name="contour_starty" type="xs:integer" use="requ
ired"/>
                        <xs:attribute name="contour_chainCode" type="xs:string" use="re
quired"/>
                        <xs:attribute name="lumenSize" type="xs:float" use="required"/>
                        <xs:attribute name="loc_x" type="xs:integer" use="required"/>
                        <xs:attribute name="loc_y" type="xs:integer" use="required"/>
                        <xs:attribute name="loc_z" type="xs:integer" use="required"/>
                        <xs:attribute name="numberOfCells" type="xs:integer" use="requi
red"/>
                        <xs:attribute name="pmax_x" type="xs:float" use="required"/>
                        <xs:attribute name="pmax_y" type="xs:float" use="required"/>
                        <xs:attribute name="pmax_z" type="xs:float" use="required"/>
                        <xs:attribute name="pmin_x" type="xs:float" use="required"/>
                        <xs:attribute name="pmin_y" type="xs:float" use="required"/>
                        <xs:attribute name="pmin_z" type="xs:float" use="required"/>
                        <xs:attribute name="polygonContour_startx" type="xs:integer" us
e="required"/>
                        <xs:attribute name="polygonContour_starty" type="xs:integer" us
e="required"/>
```

```
                        <xs:attribute name="polygonContour_chainCode" type="xs:string"
use="required"/>
                        <xs:attribute name="validation" type="xs:boolean" use="required
"/>
                        <xs:attribute name="channelindex" type="xs:integer" use="requir
ed"/>
                </xs:complexType>
                <xs:key name="MCEID">
                        <xs:selector xpath=".//MCE"/>
                        <xs:field xpath="@id"/>
                </xs:key>
        </xs:element>
        <xs:element name="SubCellular">
                <xs:complexType>
                        <xs:sequence>
                                <xs:element ref="Intensity_NVP" minOccurs="0"/>
                                <xs:element ref="Geometric_NVP" minOccurs="0"/>
                                <xs:element ref="MeanCompartment_NVP" minOccurs="0"/>
                                <xs:element ref="Organization_NVP" minOccurs="0"/>
                                <xs:element ref="Position_NVP" minOccurs="0"/>
                                <xs:element ref="Texture" minOccurs="0"/>
                                <xs:element ref="Probe" minOccurs="0" maxOccurs="unboun
ded"/>
                                <xs:element ref="SubCellularProjection" minOccurs="0" m
axOccurs="unbounded"/>
                        </xs:sequence>
                        <xs:attribute name="subc_type" type="xs:string"/>
                        <xs:attribute name="subc_computation_type" type="xs:string"/>
                        <xs:attribute name="polygonContour_starty" type="xs:integer" us
e="required"/>
                        <xs:attribute name="polygonContour_startx" type="xs:integer" us
e="required"/>
                        <xs:attribute name="polygonContour_chainCode" type="xs:string"
use="required"/>
                        <xs:attribute name="subc_id" type="xs:integer" use="required"/>
                        <xs:attribute name="contour_chainCode" type="xs:string" use="re
quired"/>
                        <xs:attribute name="contour_startx" type="xs:integer" use="requ
ired"/>
                        <xs:attribute name="contour_starty" type="xs:integer" use="requ
ired"/>
                        <xs:attribute name="validation" type="xs:boolean" use="required
"/>
                        <xs:attribute name="loc_x" type="xs:integer" use="required"/>
                        <xs:attribute name="loc_y" type="xs:integer" use="required"/>
                        <xs:attribute name="loc_z" type="xs:integer" use="required"/>
                        <xs:attribute name="pmin_x" type="xs:integer" use="required"/>
                        <xs:attribute name="pmin_y" type="xs:integer" use="required"/>
                        <xs:attribute name="pmin_z" type="xs:integer" use="required"/>
                        <xs:attribute name="pmax_x" type="xs:integer" use="required"/>
                        <xs:attribute name="pmax_y" type="xs:integer" use="required"/>
                        <xs:attribute name="pmax_z" type="xs:integer" use="required"/>
                        <xs:attribute name="radialIntensity" type="xs:string" use="opti
onal">
                                <xs:annotation>
                                        <xs:documentation>tab-delimited floats</xs:docu
mentation>
                                </xs:annotation>
                        </xs:attribute>
                </xs:complexType>
                <xs:key name="SubCellularID">
                        <xs:selector xpath=".//SubCellular"/>
                        <xs:field xpath="@subc_id"/>
```

```
                </xs:key>
        </xs:element>
        <xs:element name="SubCellularProjection">
                <xs:complexType>
                        <xs:sequence>
                                <xs:element ref="Intensity_NVP" minOccurs="0"/>
                                <xs:element ref="MeanCompartment_NVP" minOccurs="0"/>
                                <xs:element ref="Texture" minOccurs="0"/>
                        </xs:sequence>
                        <xs:attribute name="targetChannelIndex" type="xs:integer" use="
required"/>
                </xs:complexType>
        </xs:element>
        <xs:element name="Probe">
                <xs:complexType>
                        <xs:sequence>
                                <xs:element ref="ProbeEvent" minOccurs="0" maxOccurs="u
nbounded"/>
                                <xs:element ref="ProbeEventAdjList" minOccurs="0" maxOc
curs="unbounded"/>
                        </xs:sequence>
                        <xs:attribute name="channelindex" type="xs:integer" use="requir
ed"/>
                </xs:complexType>
        </xs:element>
        <xs:element name="ProbeEvent">
                <xs:complexType>
                        <xs:sequence>
                                <xs:element ref="Intensity_NVP" minOccurs="0" maxOccurs
="unbounded"/>
                                <xs:element ref="ProbeEventProjection" minOccurs="0" ma
xOccurs="unbounded"/>
                        </xs:sequence>
                        <xs:attribute name="dist_to_nuc" type="xs:float"/>
                        <xs:attribute name="contour_startx" type="xs:integer" use="requ
ired"/>
                        <xs:attribute name="contour_starty" type="xs:integer" use="requ
ired"/>
                        <xs:attribute name="contour_chainCode" type="xs:string" use="re
quired"/>
                        <xs:attribute name="probeevent_id" type="xs:integer" use="requi
red"/>
                        <xs:attribute name="loc_x" type="xs:integer" use="required"/>
                        <xs:attribute name="loc_y" type="xs:integer" use="required"/>
                        <xs:attribute name="loc_z" type="xs:integer" use="required"/>
                        <xs:attribute name="volume" type="xs:float" use="required"/>
                        <xs:attribute name="pmax_x" type="xs:float" use="required"/>
                        <xs:attribute name="pmax_y" type="xs:float" use="required"/>
                        <xs:attribute name="pmax_z" type="xs:float" use="required"/>
                        <xs:attribute name="pmin_x" type="xs:float" use="required"/>
                        <xs:attribute name="pmin_y" type="xs:float" use="required"/>
                        <xs:attribute name="pmin_z" type="xs:float" use="required"/>
                        <xs:attribute name="uncertainty" type="xs:float" use="required"
/>
                        <xs:attribute name="validation" type="xs:boolean" use="required
"/>
                </xs:complexType>
                <xs:key name="ProbeEventID">
                        <xs:selector xpath="./ProbeEvent"/>
                        <xs:field xpath="@probe_eventid"/>
                </xs:key>
        </xs:element>
        <xs:element name="ProbeEventProjection">
```

```
                <xs:complexType>
                        <xs:sequence>
                                <xs:element ref="Intensity_NVP" minOccurs="0" maxOccurs
="unbounded"/>
                        </xs:sequence>
                        <xs:attribute name="targetchannelindex" type="xs:integer" use="
required"/>
                </xs:complexType>
        </xs:element>
        <xs:element name="SubCellularAdjList">
                <xs:complexType>
                        <xs:sequence>
                                <xs:element ref="SubCellularAdjListRow" minOccurs="0" m
axOccurs="unbounded"/>
                        </xs:sequence>
                </xs:complexType>
        </xs:element>
        <xs:element name="SubCellular_adjTo">
                <xs:complexType>
                        <xs:attribute name="target_id" type="xs:integer" use="required"
/>
                </xs:complexType>
        </xs:element>
        <xs:element name="SubCellularAdjListRow">
                <xs:complexType>
                        <xs:sequence>
                                <xs:element ref="SubCellular_adjTo" minOccurs="0" maxOc
curs="unbounded"/>
                        </xs:sequence>
                        <xs:attribute name="src_id" type="xs:integer" use="required"/>
                </xs:complexType>
        </xs:element>
        <xs:element name="ProbeEventAdjList">
                <xs:complexType>
                        <xs:sequence>
                                <xs:element ref="ProbeEventAdjListRow" minOccurs="0" ma
xOccurs="unbounded"/>
                        </xs:sequence>
                </xs:complexType>
        </xs:element>
        <xs:element name="ProbeEventAdjListRow">
                <xs:complexType>
                        <xs:sequence>
                                <xs:element ref="ProbeEvent_adjTo" minOccurs="0" maxOcc
urs="unbounded"/>
                        </xs:sequence>
                        <xs:attribute name="src_id" type="xs:integer" use="required"/>
                </xs:complexType>
        </xs:element>
        <xs:element name="ProbeEvent_adjTo">
                <xs:complexType>
                        <xs:attribute name="target_id" use="required"/>
                </xs:complexType>
        </xs:element>
        <xs:element name="TextureRow">
                <xs:complexType>
                        <xs:attribute name="hist" type="xs:float" use="required"/>
                        <xs:attribute name="mean" type="xs:float" use="required"/>
                        <xs:attribute name="sd" type="xs:float" use="required"/>
                        <xs:attribute name="id" use="required"/>
                </xs:complexType>
        </xs:element>
        <xs:element name="Texture">
```

```
                <xs:complexType>
                        <xs:sequence>
                                <xs:element ref="TextureRow" minOccurs="0" maxOccurs="u
nbounded"/>
                        </xs:sequence>
                </xs:complexType>
        </xs:element>
        <xs:element name="ECM">
                <xs:complexType>
                        <xs:sequence>
                                <xs:element ref="Geometric_NVP" minOccurs="0"/>
                                <xs:element ref="Intensity_NVP" minOccurs="0"/>
                                <xs:element ref="Texture" minOccurs="0"/>
                        </xs:sequence>
                        <xs:attribute name="channelindex" type="xs:integer" use="requir
ed"/>
                        <xs:attribute name="mceID" type="xs:integer" use="required"/>
                        <xs:attribute name="id" type="xs:integer" use="required"/>
                        <xs:attribute name="pmin_x" type="xs:integer" use="required"/>
                        <xs:attribute name="pmin_y" type="xs:integer" use="required"/>
                        <xs:attribute name="pmin_z" type="xs:integer" use="required"/>
                        <xs:attribute name="pmax_x" type="xs:integer" use="required"/>
                        <xs:attribute name="pmax_y" type="xs:integer" use="required"/>
                        <xs:attribute name="pmax_z" type="xs:integer" use="required"/>
                        <xs:attribute name="contour_startx" type="xs:integer" use="requ
ired"/>
                        <xs:attribute name="contour_starty" type="xs:integer" use="requ
ired"/>
                        <xs:attribute name="contour_chainCode" type="xs:string" use="re
quired"/>
                        <xs:attribute name="polygoncontour_startx" type="xs:integer" us
e="required"/>
                        <xs:attribute name="polygoncontour_starty" type="xs:integer" us
e="required"/>
                        <xs:attribute name="polygoncontour_chainCode" type="xs:string" 
use="required"/>
                        <xs:attribute name="matrixname" type="xs:string"/>
                        <xs:attribute name="matrixtype" type="xs:string">
                                <xs:annotation>
                                        <xs:documentation>need</xs:documentation>
                                </xs:annotation>
                        </xs:attribute>
                </xs:complexType>
        </xs:element>
        <xs:element name="ImageContour">
                <xs:complexType>
                        <xs:attribute name=" channelindex" type="xs:integer" use="requi
red"/>
                        <xs:attribute name="contour_startx" type="xs:integer" use="requ
ired"/>
                        <xs:attribute name="contour_starty" type="xs:integer" use="requ
ired"/>
                        <xs:attribute name="contour_chainCode" type="xs:string" use="re
quired"/>
                        <xs:attribute name="isContourFromComposite" type="xs:boolean" u
se="required"/>
                </xs:complexType>
        </xs:element>
        <xs:element name="ImageFileSet">
                <xs:complexType>
                        <xs:sequence maxOccurs="unbounded">
                                <xs:element ref="ImageFile"/>
                        </xs:sequence>
```

```
                </xs:complexType>
        </xs:element>
        <xs:element name="ImageFile">
                <xs:complexType>
                        <xs:attribute name="sha1sum_icsHeaderFile" type="xs:string"/>
                        <xs:attribute name="dir" type="xs:string" use="required"/>
                        <xs:attribute name="filename" type="xs:string" use="required"/>
                        <xs:attribute name="sha1sum" type="xs:string" use="required">
                                <xs:annotation>
                                        <xs:documentation>If this is for ICS, this will
 be the sha1sum for the .ids file.The sha1sum for the the .ics file can be optionally r
ecorded in the sha1sum_icsHeaderFile attribute.</xs:documentation>
                                </xs:annotation>
                        </xs:attribute>
                </xs:complexType>
        </xs:element>
        <xs:element name="Cell">
                <xs:complexType>
                        <xs:sequence>
                                <xs:element ref="Intensity_NVP" minOccurs="0"/>
                                <xs:element ref="Geometric_NVP" minOccurs="0"/>
                                <xs:element ref="MeanCompartment_NVP" minOccurs="0"/>
                                <xs:element ref="Organization_NVP" minOccurs="0"/>
                                <xs:element ref="Position_NVP" minOccurs="0"/>
                                <xs:element ref="Texture" minOccurs="0"/>
                                <xs:element ref="SubCellular" minOccurs="0" maxOccurs="
unbounded"/>
                                <xs:element ref="SubCellularAdjList" minOccurs="0"/>
                        </xs:sequence>
                        <xs:attribute name="validation" type="xs:boolean" use="required
"/>
                        <xs:attribute name="pmin_z" type="xs:integer" use="required"/>
                        <xs:attribute name="pmin_y" type="xs:integer" use="required"/>
                        <xs:attribute name="pmin_x" type="xs:integer" use="required"/>
                        <xs:attribute name="cell_type" type="xs:string" use="required"/
>
                        <xs:attribute name="pmax_z" type="xs:integer" use="required"/>
                        <xs:attribute name="pmax_y" type="xs:integer" use="required"/>
                        <xs:attribute name="pmax_x" type="xs:integer" use="required"/>
                        <xs:attribute name="loc_z" type="xs:integer" use="required"/>
                        <xs:attribute name="loc_y" type="xs:integer" use="required"/>
                        <xs:attribute name="loc_x" type="xs:integer" use="required"/>
                        <xs:attribute name="contour_starty" type="xs:integer" use="requ
ired"/>
                        <xs:attribute name="contour_startx" type="xs:integer" use="requ
ired"/>
                        <xs:attribute name="contour_chainCode" type="xs:string" use="re
quired"/>
                        <xs:attribute name="polygonContour_startx" type="xs:integer" us
e="required"/>
                        <xs:attribute name="polygonContour_chainCode" type="xs:string"
use="required"/>
                        <xs:attribute name="polygonContour_starty" type="xs:integer" us
e="required"/>
                        <xs:attribute name="cell_id" type="xs:integer" use="required"/>
                </xs:complexType>
        </xs:element>
        <xs:element name="CellAdjList">
                <xs:complexType>
                        <xs:sequence>
                                <xs:element ref="CellAdjListRow" minOccurs="0" maxOccur
s="unbounded"/>
                        </xs:sequence>
```

```
				</xs:complexType>
		</xs:element>
		<xs:element name="CellAdjListRow">
				<xs:complexType>
						<xs:sequence>
								<xs:element ref="Cell_adjTo" minOccurs="0" maxOccurs="u
nbounded"/>
						</xs:sequence>
						<xs:attribute name="src_id" type="xs:integer" use="required"/>
				</xs:complexType>
		</xs:element>
		<xs:element name="Cell_adjTo">
				<xs:complexType>
						<xs:attribute name="target_id" type="xs:integer" use="required"
/>
				</xs:complexType>
		</xs:element>
		<xs:element name="ProcessingParameterSet">
				<xs:complexType>
						<xs:sequence>
								<xs:element ref="NVProp" minOccurs="0" maxOccurs="unbou
nded"/>
						</xs:sequence>
						<xs:attribute name="channel"/>
						<xs:attribute name="method"/>
				</xs:complexType>
		</xs:element>
		<xs:element name="TextureParameters">
				<xs:complexType>
						<xs:sequence>
								<xs:element ref="TextureParametersRow" minOccurs="0" ma
xOccurs="unbounded"/>
						</xs:sequence>
				</xs:complexType>
		</xs:element>
		<xs:element name="TextureParametersRow">
				<xs:complexType>
						<xs:attribute name="id" type="xs:int" use="required"/>
						<xs:attribute name="differentialOrder" type="xs:integer" use="r
equired"/>
						<xs:attribute name="phi" type="xs:float" use="required"/>
						<xs:attribute name="theta" type="xs:float" use="required"/>
						<xs:attribute name="sigma_z" type="xs:float" use="required"/>
						<xs:attribute name="sigma_y" type="xs:float" use="required"/>
						<xs:attribute name="sigma_x" type="xs:float" use="required"/>
				</xs:complexType>
		</xs:element>
</xs:schema>
```
