# Supplementary material for: BioSig3D: High Content Screening of Three-Dimensional Cell Culture Models
Source: PLoS One. 2016 Mar 15;11(3):e0148379. doi: 10.1371/journal.pone.0148379 (PMC4792475; doi:10.1371/journal.pone.0148379)
Supplement: S4 Text — (DOCX) [file pone.0148379.s004.docx]

**S4 Text: Colony organization of computed indices**

| 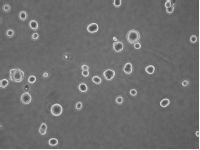 | 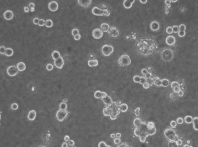 | 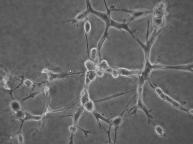 |
| --- | --- | --- |
| (a) | (b) | (c) |

**Fig A in S4 Text:** Genetic defects lead to distinct colony organization in each class of the breast cancer cell lines: (a) mass, which is ER+, PR+, and ERBB2 negative; (b) grape-like, which is generally ERBB2 positive with EGFR amplification, and (c) triple negative. Each blob consists of a number of cells that are organized to form a colony.

Table A in S4 Text: Computed global shape indices for colony formation

| Size | Number of pixels in all nuclei within the colony. |
| --- | --- |
| PhysicalSize | The physical size (e.g., in micron) of all nuclei within a colony. |
| EquivalentSphericalRadius | The radius of the sphere, which is the same size as the colony, i.e., fitting an equal volume sphere to the colony. |
| EquivalentSphericalSurface | The surface area of the sphere, which is the same size as the colony. |
| EquivalentEllipsoidDiameterX, Y, Z | The diameter of the ellipsoid in X,Y,Z direction, which has the same size as the colony. |
| PrincipalMomentsX, Y, Z | Computed from the aggregate nuclear masks of the colony, based on the eigenvalues of the moment’s matrix. |
| Elongation | The ratio of the largest principal moment to the smallest principal moment. |
| Flatness | The ratio of the largest principal moment to the second smallest principal moment. |
| Roundness | Deviation from a spherical shaped object. |
| Perimeter | In 2D the circumference of the object, in 3D the area of the object. |
| Radius | Radius is based on *minimum eccentricity* computed from a Delaunay triangulation from centroids of nuclei in a colony. Eccentricity is defined as the maximum graph distance between one vertex and all other vertices in a graph. |
| Diameter | Diameter is based on *maximum eccentricity* computed from a Delaunay triangulation from centroids of nuclei in a colony. Maximum eccentricity is defined as the “longest shortest path” between any two graph vertices. |
| ConvexHullVolume | Volume of convex hull that is fitted to all nuclei within a colony. |

Table B in S4 Text: Computed geometric indices profiling local organization from a graph generated through Delaunay Triangulation

| LumenVolume | Normal mammary epithelial cells will form a hollow sphere (e.g., a lumen). Lumen volume index is computed as a function of the volume of the convex hull minus the volume occupied by the segmented nuclei. |
| --- | --- |
| Clustering Coefficient of a Node | The degree to which vertices in the graph tend to co-cluster. |
| MeanClusteringCoefficient | The average clustering coefficient for the graph. |
| MeanCloseness | Average of the total graph-theoretic distance from one node to all other nodes in the graph. |
| MeanDegree | Degree of a vertex u is the number of vertices that are adjacent to u. |
| MeanGeodesicDistance | The average distance between any two vertices in the graph. |
| Eccentricity of a Node | Maximum value of the shortest path from a given node to any other node. |
| MeanEccentricity | Average eccentricity over all the nodes. |
| Closeness of a Node | Average value of the shortest path from a given node to any other node. |
| Girth | The length of the shortest cycle in the graph |
| Circumference | The length of the longest cycle in the graph. |
| NumberOfConnectedComponents | The total number of disconnected components in the graph. |
| MeanComponentSize | The average number of vertices of the connected components. |
| GiantComponentRatio | Ratio of the size of the largest set of the nodes that are reachable from each other to the number of nodes. |
| MaximumCliqueSize | A clique (e.g., subgraph) with maximum number of vertices. |
| NumberOfEdges | Number of edges in the graph. |
| NumberOfVertices | Number of cells in the tissue. |
| MinEdgeLength | The physical length of the minimum edge present in the graph. |
| MaxEdgeLength | The physical length of the maximum edge present in the graph. |
| MeanEdgeLength | Average physical length of an edge in the graph. |
| StdEdgeLength | Second order statistics of the edge length distribution. |
| SkewnessEdgeLength | Third order statistics of the edge length distribution. |
| KurtosisEdgeLength | Fourth order statistics of the edge length distribution. |
| MSTMinEdgeLength | The length of the minimum edge length computed from the minimum spanning tree of the colony. |
| MSTMaxEdgeLength | The length of the maximum edge length computed from the minimum spanning tree of the colony. |
| MSTMeanEdgeLength | The average length of the edges computed from the minimum spanning tree of the colony. |
| MSTStdEdgeLength | Standard deviation computed from the edge length of the minimum spanning tree. |
| MSTSkewnessEdgeLength | Skewness computed from the third moment of the distribution of the “edge lengths” of the minimum spanning tree. |
| MSTKurtosisEdgeLength | Kurtosis computed from the fourth moment of the distribution of the “edge lengths” of the minimum spanning tree. |
| MSTPercentOfEndPoints | The ratio of number of vertices that have only one edge (e.g., a leaf node) to the total number vertices in the minimum spanning tree. |
| MSTPercentOfBranchPoints | The ratio of the number of vertices that have more than 2 edges to the total number vertices in the minimum spanning tree. |
